# Characteristics and pH-Responsiveness of SDBS–Stabilized Crude Oil/Water Nanoemulsions

**DOI:** 10.3390/nano12101673

**Published:** 2022-05-13

**Authors:** Sagheer A. Onaizi

**Affiliations:** Department of Chemical Engineering, Interdisciplinary Research Center for Hydrogen and Energy Storage, King Fahd University of Petroleum and Minerals, Dhahran 31216, Saudi Arabia; onaizi@kfupm.edu.sa

**Keywords:** crude oil-in-water (O/W) nanoemulsions, pH-responsive demulsification mechanism, rheology, droplet size, zeta potential, interfacial tension

## Abstract

Nanoemulsions are colloidal systems with a wide spectrum of applications in several industrial fields. In this study, crude oil-in-water (O/W) nanoemulsions were formulated using different dosages of the anionic sodium dodecylbenzenesulfonate (SDBS) surfactant. The formulated nanoemulsions were characterized in terms of emulsion droplet size, zeta potential, and interfacial tension (IFT). Additionally, the rheological behavior, long-term stability, and on-demand breakdown of the nanoemulsions via a pH-responsive mechanism were evaluated. The obtained results revealed the formation of as low as 63.5 nm average droplet size with a narrow distribution (33–142 nm). Additionally, highly negative zeta potential (i.e., −62.2 mV) and reasonably low IFT (0.45 mN/m) were obtained at 4% SDBS. The flow-ability of the nanoemulsions was also investigated and the obtained results revealed an increase in the nanoemulsion viscosity with increasing the emulsifier content. Nonetheless, even at the highest SDBS dosage of 4%, the nanoemulsion viscosity at ambient conditions never exceeded 2.5 mPa·s. A significant reduction in viscosity was obtained with increasing the nanoemulsion temperature. The formulated nanoemulsions displayed extreme stability with no demulsification signs irrespective of the emulsifier dosage even after one-month shelf-life. Another interesting and, yet, surprising observation reported herein is the pH-induced demulsification despite SDBS not possessing a pH-responsive character. This behavior enabled the on-demand breakdown of the nanoemulsions by simply altering their pH via the addition of HCl or NaOH; a complete and quick oil separation can be achieved using this simple and cheap demulsification method. The obtained results reveal the potential utilization of the formulated nanoemulsions in oilfield-related applications such as enhanced oil recovery (EOR), well stimulation and remediation, well-bore cleaning, and formation fracturing.

## 1. Introduction

Nanoemulsions are colloidal systems where a liquid is dispersed in another immiscible liquid as fine droplets having an average size below 500 nm [1]. These nano-dispersions (or nano-fluids) can be water-in-oil (W/O), oil-in-water (O/W), or multiple (complex) emulsions (i.e., O/W/O or W/O/W). The dispersion of one of the two immiscible liquids in another is obtained using different techniques such as phase inversion, ultrasonication, microfluidization, and homogenization methods [2]. To achieve kinetically stable emulsions, surfactants, which are surface active molecules with several applications [3,4,5,6,7,8,9,10,11,12], are added during the emulsification process. Depending on the hydrophilic/lipophilic balance (HLB) of the utilized surfactant, W/O (when HLB is ≤6) or O/W (when HLB is ≥8) can be formulated. Complex emulsions can be obtained when at least two surfactants with high and low HLB values are used [13]. The characteristics (droplet size of the dispersed phase, interfacial tension, zeta potential, etc.) of the formulated emulsions depend on factors such as the nature and concentration of the utilized surfactant, the characteristics of both the dispersed and the continuous phase, and the preparation method. Emulsions with small droplet size (nano-sized), low interfacial tension (IFT), and high absolute zeta potential tend to be more kinetically stable [14,15]; long-term emulsion stability is highly desirable when emulsions with a long-shelf life are required. 

Nanoemulsions are widely applied across a number of industries such as food and cosmetics [16,17], pharmaceutical and medical applications [18,19], and oilfield-related industries [1,20], among other applications. Although the application of nanoemulsions in oilfield endeavors is relatively new, it is gaining significant momentum. Nanoemulsions have the potential to reduce IFT and, accordingly, the capillary pressure of the reservoir [1], improve rock wettability [1], ease the fracturing fluid flow-back [21], eliminate phase trapping [21,22], and increase the regained gas permeability [1,21]. Accordingly, some researchers have recently demonstrated the applicability of nanoemulsions in enhanced oil recovery (EOR) [23,24,25], production enhancement (e.g., well stimulation and remediation, well-bore cleaning, and formation fracturing) [26,27,28], and pumping of extra heavy crude oil [29]. For instance, it has been reported that nanoemulsions can (i) minimize/control water-cut [28], (ii) create new channels for gas flow (i.e., increase gas permeability) [28], (iii) increase proppant permeability and, accordingly, lower the initial cleanup pressure [30], (iv) reduce fingering [31], (v) enhance the flow-back rates of fracturing fluids [31], (vi) reduce/eliminate water blocking problems [27], and (vii) minimize surfactant adsorption to the reservoir rocks [1], among other beneficial effects. 

Accordingly, it has been stated that nanoemulsions can revolutionize the oil production industry once properly utilized [1]. Certainly, one the key factors for utilizing nanoemulsions for oilfield as well as other applications is the formulation of extremely stable nanoemulsions. There are a number of studies on the formulation of nanoemulsions; however, most of the proposed emulsions contain solid particles (i.e., Pickering emulsions). Although the investigated Pickering nanoemulsions showed promising results, the potential aggregation of the fine solid particles into bigger flocs/aggregates larger than the pore size (can be as small as 100 nm [1]) should not be overlooked. The risk of forming flocs/aggregates that are bigger in size than the reservoir pores could lead to the permanent pore clogging. Therefore, the development of solid-free nanoemulsions seems more appropriate for EOR applications. Solid-free nanoemulsions can be formulated using surfactants, polymers and alkali; however, the presence of big polymeric molecules might plug the reservoir pores/capillaries, resulting in low permeability formations [32,33]. Additionally, the presence of alkali might lead to scale formation and potentially irreversible damages to the reservoir when alkalis are combined with polymers [34]. Accordingly, the ability to formulate nanoemulsions with attractive characteristics and favorable rheological properties using merely surfactants (particularly, using a single surfactant that is commercially available and relatively cheap such as SDBS, for instance) is very attractive. Additionally, having nanoemulsions that are easily switchable will be even more appealing [6,14,35,36,37].

Therefore, this work focuses on the formulation and stabilization of crude oil/water nanoemulsions using SDBS. The formulated O/W nanoemulsions were characterized (at different SDBS dosages) in terms of average droplet size (as well as size distribution), the ability of the nanoemulsion to reduce the tension of diesel/nanoemulsion interface, and the zeta potential of the nanoemulsion droplet. The reason for using diesel as the surrounding oil phase instead of the crude oil in the IFT studies using the drop shape analysis technique is the opacity of the crude oil. In addition to the above instigations, the flow behavior of the formulated nanoemulsions under different temperatures was also studied in this work. Additionally, the long-term (>30 days) kinetic stability of the prepared nanoemulsions was also investigated herein. Furthermore, the ability to destabilize the extremely stable O/W nanoemulsions by simply adjusting their pH through HCl or NaOH addition was also investigated in this study. Despite the utilization of SDBS in a number of applications, there is still a great lack of information in the published literature on similar systems (e.g., crude oil/water/SDBS), particularly, the long-term emulsion stability and the pH-induced emulsion breakdown. The demulsification of other emulsions stabilized by emulsifiers that lack the pH-responsiveness character, as is the case with SDBS, via the pH-alteration mechanism is also still lacking in the published literature, to the best of our knowledge. 

## 2. Materials and Methods

### 2.1. Materials

Crude oil containing 5.5 wt% resins, 16.2 wt% asphaltenes, 42.2 wt.% saturates, and 36.1 wt% aromatics with an API gravity of 36.4° was obtained locally while SDBS was obtained from Sigma-Aldrich (Rockville, MD, USA). Reagents used in this work were of analytical grade purity; the reagents were also obtained from Sigma-Aldrich.

### 2.2. Nanoemulsion Formulation

The initial step in the preparation of the crude oil/water nanoemulsions was the mixing of the emulsion ingredients (i.e., crude oil, water, and SDBS) by stirring the mixture overnight at room temperature. Then, the coarse emulsion was subjected to a 15 min sonication, yielding the nanoemulsion. To avoid emulsion overheating during sonication, the container containing the emulsion was soaked in a cold-water bath. The volumes of water and crude oil were kept constant at 80 and 20 vol%, respectively, while the emulsifier content (i.e., SDBS) was varied at 0.1, 0.5, 2, and 4 wt% (i.e., weight of SDBS/volume of the liquid phase × 100%). The type of the formed nanoemulsions was confirmed using the solubility test where drops were withdrawn from the obtained emulsions and placed in water and diesel. The drops placed in diesel remained intact while those placed in water mixed with water and disappeared, which confirms the formation of O/W emulsions. 

### 2.3. Long-Term Stability of the Formed Nanoemulsions 

In order to evaluate the long-term stability of the formulated nanoemulsions, the common bottle test method was utilized. This was done by placing a specific volume (e.g., 10 mL) of each emulsion in a graduated cylinder, that was tightly closed, and measuring the separated crude oil or/and water volume (if any) over a period of at least one month. From the obtained values of the time-dependent separated crude oil (or water) volume (if any), the demulsification (i.e., the percentage of the separated crude oil (or water) relative to the original crude oil/water in the emulsion) was calculated and plotted against time.

### 2.4. Characteristics of the Formed Nanoemulsions 

Average droplet size and droplet size distribution, IFT between the emulsion and an oil phase (e.g., diesel), and zeta potential of the formed emulsions are very important emulsion characteristics. In order to get quantitative insights into these characteristics, the following measurements were conducted.

#### 2.4.1. Droplet Size Measurements 

In these measurements, the light scattering technique was utilized to measure the droplet size and size distribution. Specifically, the Zetasizer Nano ZS90 (Malvern Instruments Ltd., Worcestershire, UK) was used. These measurements were conducted immediately after the nanoemulsion preparation in order to avoid/minimize the droplet coalescence effect (if any) on the measured droplet size and size distribution.

#### 2.4.2. Zeta Potential Measurements

The zeta potential of the formulated nanoemulsions was also measured using Zetasizer Nano ZS90 instrument. These measurements were also conducted immediately once the sonication process (i.e., the second step of the nanoemulsion preparation) was completed. 

#### 2.4.3. Interfacial Tension Measurements

Interfacial tension between the formulated nanoemulsions and diesel was measured using a drop shape analysis technique (DSA25, KRÜSS, Hamburg, Germany). In each measurement, an emulsion drop was first placed in a needle, then, the tip of the needle (with the pendant emulsion drop) was immersed in a quartz cuvette filled with diesel. The changes in the drop shape were converted into IFT using ADVANCE 1.8 Software (KRÜSS, Hamburg, Germany). The changes in IFT were recorded for 1 h and the final IFT value was taken as the equilibrium one. 

### 2.5. Rheology of the Formulated Nanoemulsions 

Rheometer HR 10 (TA Instruments, Waters GmbH, Eschborn, Germany) was used to conduct the rheological tests. In these tests, the apparent viscosity of each of the formulated nanoemulsions was measured over a wide temperature range (from 25 up to 85 °C). In these measurements, the shearing rate was fixed at 100 s^−1^. 

### 2.6. Demulsification Induced by pH Alteration

Although SDBS does not possess pH-responsiveness character, we hypothesize that its hydrolysis [38,39,40] and/or interaction with the surface active components of the crude oil [14] might render the system pH-responsive, at least to a certain degree. Therefore, immediately after the preparation of the crude O/W nanoemulsions, their pH was switched to a higher or a lower value through the addition of NaOH or HCl, then, the demulsification was measured following the bottle test method mentioned above. Specifically, 9 mL of each of the freshly prepared nanoemulsions was placed in a graduated cylinder, then 1 mL of either NaOH or HCl (final concentration of NaOH or HCl in the emulsion was 0.5 M) was gently dispersed in the emulsion (no shaking or any other external disturbances were introduced). Then, the cylinder was tightly closed and kept at room temperature for more than 30 days. At different time intervals, readings of the separated crude oil or/and water volume (if any) from the emulsion were taken and the demulsification was calculated as mentioned previously. 

## 3. Results and Discussion

Rheological properties of emulsions are key factors in a number of applications. However, before presenting the results of the rheological studies, some important emulsion characteristics (i.e., droplet size, zeta potential, and interfacial tension) are presented and discussed. 

### 3.1. Average Droplet Size and Size Distribution 

**Figure 1:**
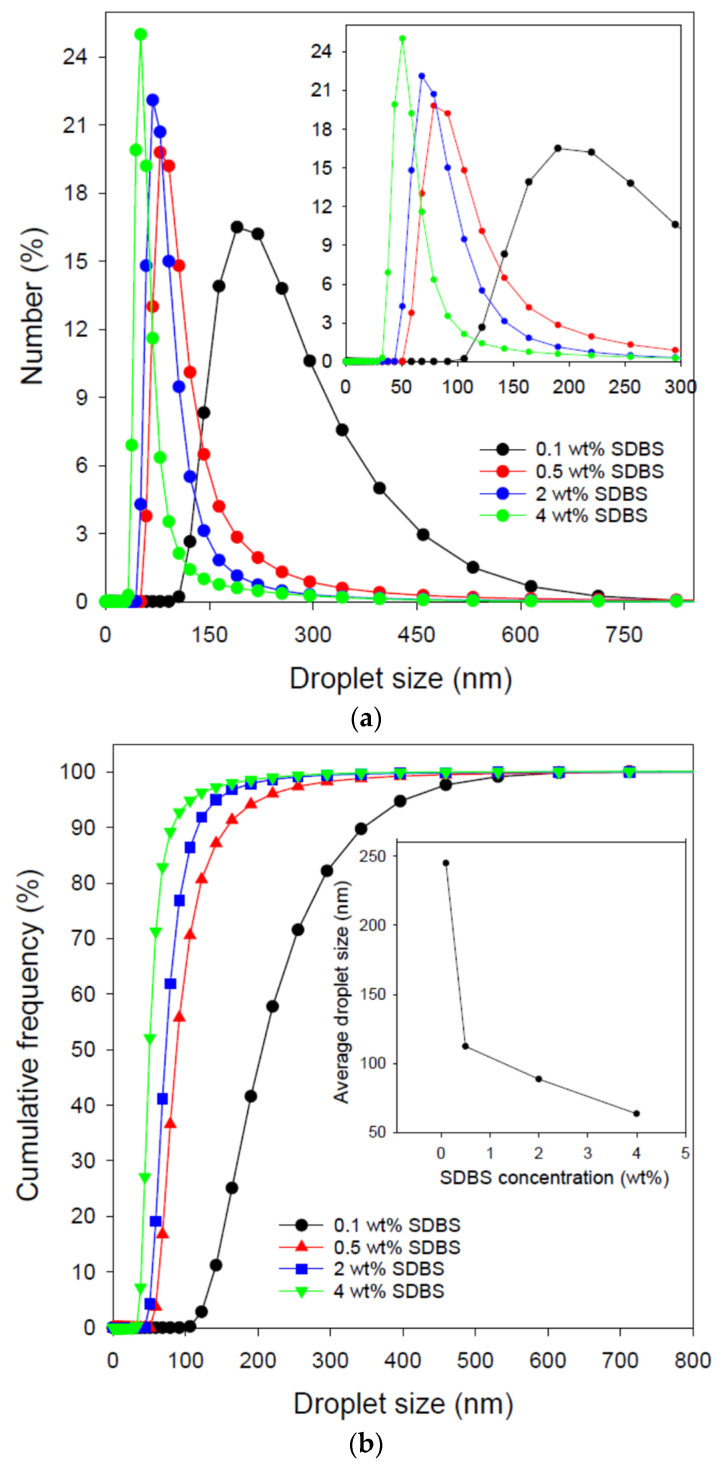
(**a**): Effect of SDBS concentration on the droplet size distribution of the formulated crude oil/water nanoemulsions. The inset shows the droplet size distribution up to 300 nm. (**b**): Effect of SDBS concentration on the cumulative frequency of droplet size. The inset shows the average droplet size at different SDBS concentrations.

As previously stated, droplet size is one of the key characteristics of an emulsion. Figure 1 shows the droplet size of the dispersed crude oil phase at different concentrations of the emulsifier (i.e., SDBS). As demonstrated in Figure 1a, all emulsions are unimodal. With increasing the emulsifier concentration, the droplet size distribution becomes narrower (i.e., 106–615, 59–295, 50–190, and 33–142 nm, at SDBS concentration of 0.1, 0.5, 2, and 4 wt%, respectively; see the cumulative plots in Figure 1b). The narrowing of the nanoemulsion droplet size with increasing SDBS concentration stems from the presence of higher populations of the emulsifier molecules during the emulsion preparation, promoting the fission of bigger droplets into smaller ones. Accordingly, the average droplet size of the crude oil/water emulsion stabilized by 0.1, 0.5, 2, and 4 wt% SDBS are 244.7, 112.3, 88.6, and 63.5 nm, respectively (see the inset of Figure 1b) due to the increase in the fraction of smaller droplets with increasing the emulsifier concentration. 

However, the relationship between the emulsifier concentration and the average droplet size is not linear (see the inset of Figure 1b). After a sharp decrease in the average droplet size with increasing the SDBS concentration from 0.1 to 0.5 wt%, a gradual decrease took place. This observation has been reported elsewhere [15,41,42]. For instance, Komaiko and McClement [41] used the nonionic surfactant Tween 80 to stabilize medium chain triglycerides (MCT) oil/water emulsion and reported a sharp decrease in the dispersed MCT droplet size with increasing the surfactant-to-oil ratio up to 1 (note: surfactant dosage was increased at a fixed a fixed oil content of 10 vol%). 

Beyond this ratio, there were only marginal changes in the emulsion droplet size with further increase the surfactant content, reaching about 100 nm at a surfactant-to-oil ration of 2. Additionally, the droplet size distribution became narrower with increasing Tween 80 concentration in the emulsion. Nonetheless, Komaiko and McClement [41] reported that Tween 80 was more effective than other Tween and Span surfactants in producing a smaller droplet size. However, such an effectiveness was compromised when different oils were used, for which the mean droplet size was as high as 10.5 µm. A decrease in droplet size with a narrower size distribution was also reported by Kumar and Mahto [42] for heavy crude oil/water emulsion stabilized by tri–triethanolamine monosunflower ester. In another study, Kumar and Mandal [15] used *n*-heptane and Tween 40 to formulate O/W nanoemulsions and reported that the emulsion droplet size decreased with increasing the surfactant concentration up to 0.5%, followed by an increase in the droplet size upon increasing the surfactant concentration further. This unusual trend has been justified by the slower release of the surfactant molecules from their micelles at higher surfactant concentrations. 

### 3.2. Zeta Potential

**Figure 2:**
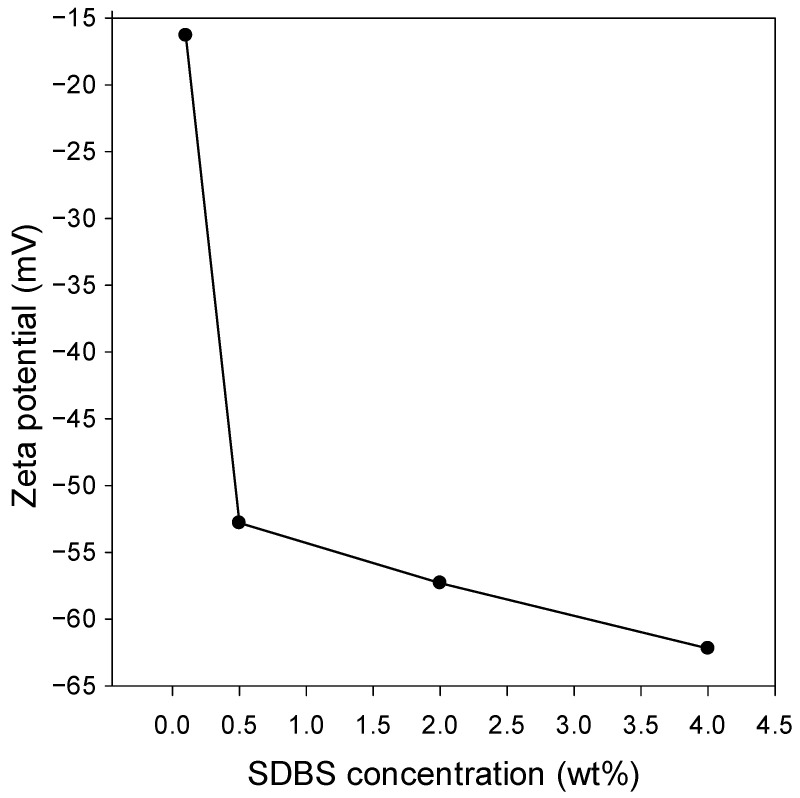
Zeta potential values of the formulated crude oil/water nanoemulsions at different concentrations of the emulsifier (i.e., SDBS).

According to the results presented in Figure 1, all the formulated crude oil/water emulsions have nanometer-sized average diameters and, thus, can be termed nanoemulsions. It has been reported that an inverse relationship between emulsion stability and droplet size exists. Accordingly, emulsions with smaller droplet sizes are usually more stable than those with bigger ones. Another factor that is indirectly related to droplet size is zeta potential. Figure 2 shows the effect of surfactant concentration on the zeta potential of the nanoemulsions. As the concentration of the anionic surfactant (i.e., SDBS) increases, the zeta potential value becomes more negative due to the presence of more surfactant anions at the droplet surface. For instance, the zeta potential drops from −16.3 to −52.8 mV upon increasing SDBS concentration from 0.1 to 0.5 wt%. Increasing SDBS concentration above 0.5 wt% resulted in a gradual decrease in zeta potential, reaching −62.2 mV at an SDBS concentration of 4 wt%. It has been proposed in the literature that colloidal systems (including emulsions) with an absolute zeta potential value greater than 30 mV are kinetically stable. Accordingly, with the exception of the crude oil/water nanoemulsion stabilized by 0.1 wt% SDBS, all the crude O/W nanoemulsions formulated in this work are expected to be stable. Nonetheless, even the one formulated using the lowest SDBS concentration (i.e., 0.1 wt%) revealed extreme stability as will be presented and discussed later.

It has been proposed that the presence of polymers enhances emulsion stability via steric hindrance effect [43]. Since crude oils contain naturally occurring polymeric components (e.g., asphathltenes and resins), the extreme stability of the nanoemulsion stabilized by 0.1 wt% SDBS, despite the low zeta potential, might stem partially from the interconnectedness between the tangling polymeric chains surrounding (encapsulating) the oil droplets. When combined with electrostatic repulsion induced by the negative charges on the SDBS ions adsorbed at the surface of the oil droplet, emulsion stability further improves [43]. 

The displayed results in Figure 2 are comparable to those obtained for other systems as reported in the literature [44,45,46,47]. For instance, SDS-stabilized paraffin wax/water emulsion displayed a zeta potential value of −38.3 mV at a surfactant concentration of 0.01 mg/mL, which slightly decreased to −38.9 mV when SDS concentration was increased by 100 folds [44]. Kumar and Mandal [45] prepared light mineral oil/water nanoemulsions, which were stabilized by the anionic surfactant polymethyl ester sulfonate. The authors reported a decrease in the zeta potential of the nanoemulsion from about −21 to −31 mV upon increasing the surfactant concentration from 0.5 to 2 wt%. Comparing the increase in the absolute value of the zeta potential reported in the abovementioned studies with the results obtained herein reveals the superiority of SDBS in boosting the emulsion absolute zeta potential. Nonetheless, Kumar and Mandal fixed the light mineral oil at 10 wt%; certainly, the oil type and concentration can affect zeta potential value. In another study, Kumar and Mandal [46] used the same oil (i.e., light mineral oil) and the nonionic Tween 40 surfactant to prepare O/W emulsions and reported zeta potential values ranging from about −30 to −40 mV using 0.5–2 wt% surfactant. Similarly, Liu et al. [47] used Tween 80 and Span 80 (both are nonionic surfactants) to stabilize a paraffin/water emulsion and observed a decrease in the zeta potential of the emulsion from about −30 to −38 mV as the surfactant concentration increased from 4 to 8 wt%. Although nonionic surfactants are not electrically conductive and should have a minimal/no effect on zeta potential of colloidal systems, it has been proposed that when an electric field is applied during zeta potential measurements, some ion pairs in the nonionic surfactants breakdown, generating electrons, which in turn affect the zeta potential value. 

### 3.3. IFT between the Formulated Nanoemulsions and Diesel

**Figure 3:**
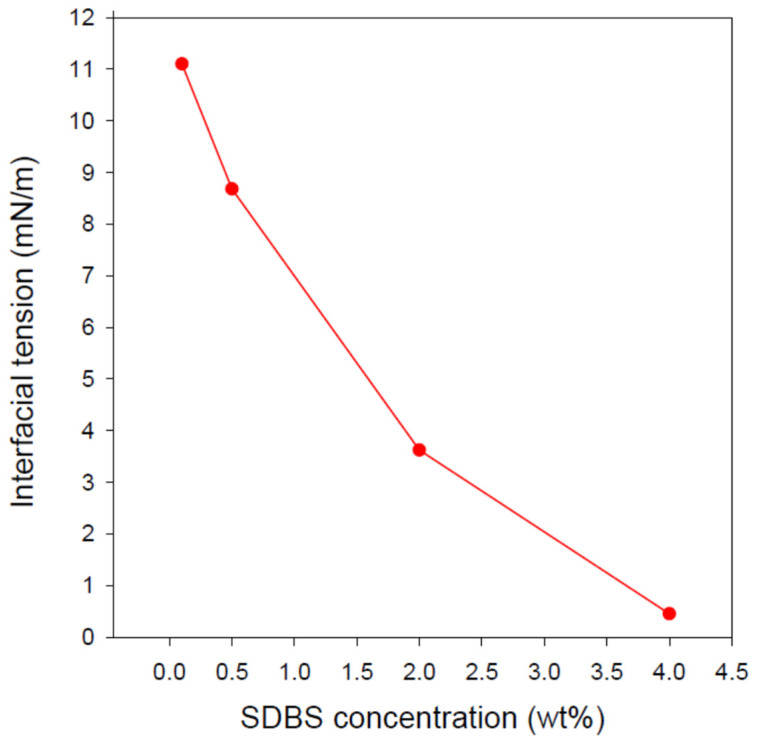
Effect of SDBS concentration on the equilibrium IFT (taken after 1 h) of the nanoemulsions. Due to the opacity of crude oil, diesel was used instead as the surrounding oil phase during the IFT measurements.

Undoubtedly, interfacial tension is an important characteristic of emulsions due to the interplay between IFT and emulsion stability as well as between these two and the emulsion rheology and other characteristics. For instance, nanoemulsions with lower IFT values are generally more kinetically stable, making them good candidates for various applications. Additionally, low IFT is usually associated with low droplet size and viscosity. Figure 3 shows the effect of SDBS concentration on the IFT between diesel (i.e., the surrounding medium) and pendant drops of the prepared O/W nanoemulsions. The IFT values displayed in Figure 3 were taken after incubating the emulsion droplets in diesel for 1 h to ensure the attainment of equilibrium. The IFT dropped from about 11.1 to 0.45 mN/m upon increasing SDBS concentration from 0.1 to 4 wt% as a result of more surfactant adsorption at the emulsion-diesel interface with increasing SDBS concentration in the emulsion. Surprisingly, despite the existence of a huge number of publications reporting IFT between aqueous phase of different surfactants and different oil phases, there is a huge lack of studies on the interfacial tension of emulsions (whether O/W or W/O) and a surrounding oil or aqueous phase. The exception, to the best of our knowledge, is the recent study published by Kumar and Mandal [46] where the IFT between *n*-heptane and an O/W emulsion droplet (prepared using the aqueous solution of Tween 40 and light mineral oil) was reported. In the above-mentioned study, the IFT between *n*-heptane and the emulsion decreased with increasing Tween 40, reaching 1.6 mN/m for the emulsion stabilized by 2 wt% Tween 40 when IFT measurements were conducted at 30 °C. Considering the recent reports, which suggest the superiority of the nanoemulsion flooding over the traditional chemical flooding in enhancing the recovery of trapped crude oil, the IFT between the emulsion/oil systems is of more relevance than simply studying the IFT between the aqueous solution of a given surfactant and an oil.

As stated in the Materials and Methods section, the IFT measurements were taken against diesel. Certainly, it would be better to measure the IFT against crude oil but due to the crude oil opacity, it was replaced with the transparent diesel oil in order to enable such measurements using the drop shape analysis technique. Looking again at Figure 3 reveals the continuous decrease in the IFT between diesel and the nanoemulsions. This is an interesting observation since it is commonly known that the decrease in the interfacial/surface tension usually levels off when the surfactant concentration exceeds its critical micelle concentration (CMC) [48,49]. Since the CMC of SDBS is below 0.1 wt% [49,50], it would be intuitively expected that increasing SDBS concentration in the studied range (0.1–4 wt%) should not result in a significant change in the IFT of the diesel/emulsion interface, which is not the case. Therefore, it seems that an increase in SDBS adsorption at the diesel/emulsion interface still occurs with increasing its concentration at least up to 4 wt% as can be inferred from the IFT results shown in Figure 3. This observation indirectly suggests that there is still a room for the SDBS molecules to attach themselves at the diesel/nanoemulsion interface. In other words, the diesel/emulsion interface is not fully packed yet and, thus, a further increase in the surfactant concentration might result in a further decrease in the IFT of the diesel/emulsion system studied herein. It is worth mentioning that Kumar and Mandal [46] also did not observe an IFT levelling off of the investigated system (i.e., Tween 40-stabilized light mineral oil/water emulsion against *n*-heptane) despite that the utilized Tween 40 concentrations were also above its CMC. 

**Figure 4:**
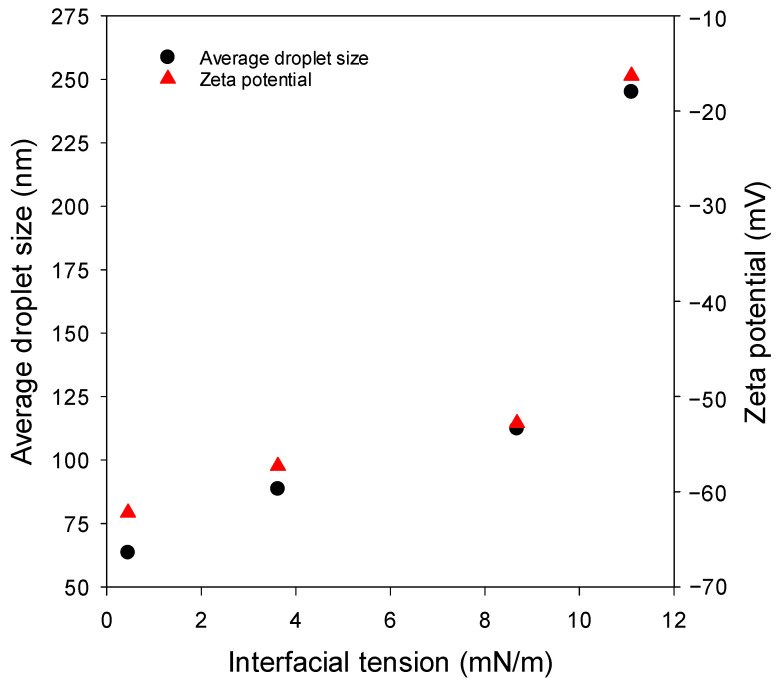
The “approximately” exponential-like correlation between IFT and the average droplet size and also between IFT and zeta potential of the formulated O/W nanoemulsions.

IFT is also directly related to the emulsifier adsorption at the interface between the emulsion droplet and the surrounding bulk of liquid. With further increase in the population of the emulsifier molecules at the interface, the interfacial density of the emulsifier will increase (higher packing density of the emulsifier molecules at the interface), resulting in a lower IFT [50,51,52,53]. Higher packing of the emulsifier molecules at the interface increases the repulsion between emulsion droplets, promoting an increased emulsion stability. Additionally, the presence of large enough molecules of the emulsifier in the aqueous solution leads to the formation of smaller droplets of the dispersed phase. Accordingly, the existence of a positive correlation between emulsion droplet size and IFT is expected. Furthermore, zeta potential and IFT are expected to be correlated. Figure 4 shows such correlations. The relationship between IFT and the average droplet size of the nanoemulsion and also between IFT and the zeta potential are both “approximately” exponential-like functions. 

### 3.4. Rheological Studies 

**Figure 5:**
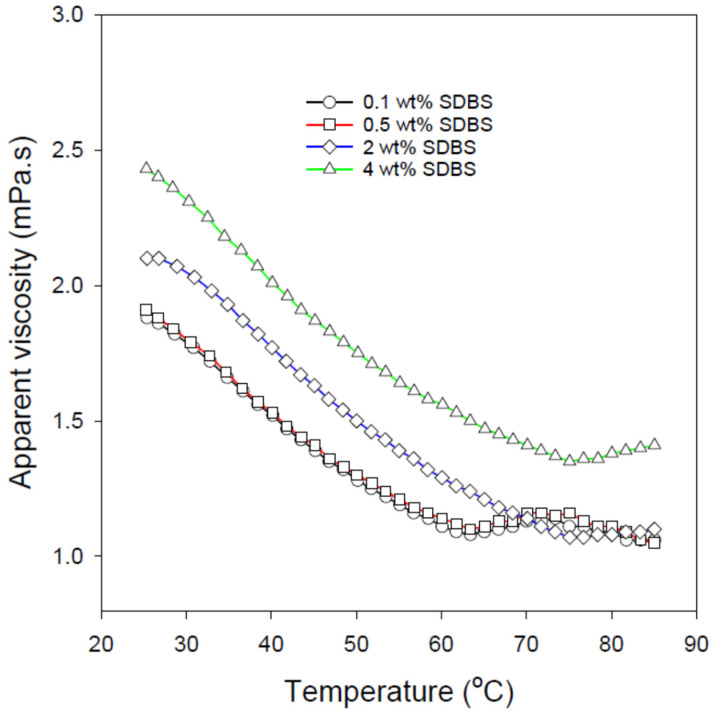
The effect of temperature on the apparent viscosity of the formulated O/W nanoemulsions using different SDBS concentrations. These measurements were conducted at 100 s^−1^ shear rate.

Viscosity (e.g., flow-ability) is one of the most important properties of colloidal systems including emulsions. Certainly, varying temperature would have an impact on the nanoemulsion viscosity. Therefore, the effect of temperature on the apparent viscosity of the formulated O/W nanoemulsions has been studied at a fixed shear rate of 100 s^−1^ and the results are depicted in Figure 5. Although the apparent viscosities of the O/W nanoemulsions are still generally proportionally related to the SDBS dosage, increasing the thermal energy of the nanoemulsions (i.e., exposing them to higher temperatures) results in a significant decrease in their viscosities. This is because the increase in the nanoemulsion temperature leads to an increase in the Brownian motion of the nanoemulsion droplets (i.e., higher droplet mobility). Higher droplet mobility results in a reduction in the interactions and the cohesive forces between neighboring droplets and, accordingly, a drop in the nanoemulsion viscosity. 

### 3.5. Emulsion Stability

Emulsion stability is governed by a number of factors such as the emulsifier concentration, oil content, the characteristics of the emulsifier, the properties of the dispersed and continuous phase, the emulsification method, etc. Considering some recent studies which pinpoint the superiority of micro/nanoemulsion flooding relative to other tertiary EOR methods [23,24,25], extreme emulsion stability is a key requirement for deploying such a new oil recovery method. According to the results presented and discussed in the previous sections, the nanoemulsions formulated in this work possess interesting characteristics such as nano-sized emulsion droplets, highly negative zeta potential, reasonably low IFT, and attractive rheological behaviors. Additionally, the results of IFT, zeta potential, and droplet size measurements suggest a good emulsion stability. Furthermore, according to the obtained results, stability would increase with increasing the emulsifier concentration. 

**Figure 6:**
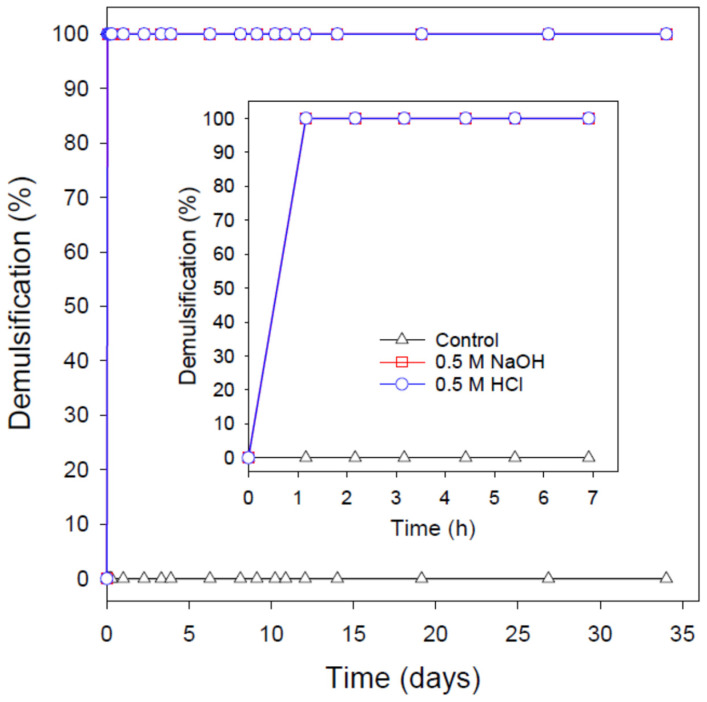
The long-term stability and demulsification process of the crude O/W nanoemulsion stabilized by 0.1 wt% SDBS using the pH-switching mechanism through the addition of 0.5 M HCl or NaOH. The demulsification process was conducted at ambient conditions and in the absence of any physical disturbances. The first 7 h of the demulsification process is shown in the inset.

In order to gain quantitative insights into the stability of the formulated crude O/W nanoemulsions, particularly the long-term one, emulsion stability tests were conducted as articulated in the Materials and Methods section. In these tests, the percentage of demulsification (i.e., oil or/and water separation) was recorded over a period of at least one month. Figure 6 shows the result of such tests for the crude O/W nanoemulsion stabilized by 0.1 wt% SDBS (i.e., the control experiment). As displayed in the abovementioned figure, this nanoemulsion is extremely stable with 0% demulsification (i.e., 100% emulsion stability) even after more than 30 days of storage. Since this nanoemulsion is stabilized by a relatively lower level of SDBS and it possesses the highest IFT and droplet size on one hand and the lowest absolute zeta potential on the other hand, it is expected to be the least stable among other nanoemulsions reported herein. Nonetheless, the long-term stability of other nanoemulsions was also monitored for at least one month and none of them showed any sign of demulsification (i.e., 0% oil and water separation). 

Although there is a huge publication volume on the short-term emulsion stability (e.g., within 24 h), the number of studies reporting mid-term (few days) or long-term stability (≥30 days) is comparatively much lower. One of the mid-term stability studies was recently published by Jia et al. [54] where SDBS was used to stabilize *n*-dodecane/water emulsion (O/W) and the prepared emulsion was stable for at least 3 days when SDBS concentration was ≥30 mM (≥1 wt%). Similarly, Kumar and Mahto [42] studied the stability of crude oil/water emulsion at different surfactant (i.e., tri–triethanolamine monosunflower ester) concentrations for 6 days. The authors reported that for O/W emulsion containing 60% oil, the stability at 25 °C increased from about 83 to 95% when the surfactant concentration was increased from 1 to 3 wt%. In another study, Kumar and Mahto [29] stabilized crude oil/water emulsions using PEG monolinoleate surfactant and reported a decline in the emulsion stability with time, reaching about 33–70% for emulsions stabilized by 0.5–2 wt% surfactant. Unlike the above studies, Jadhav et al. [44] reported that paraffin wax/water emulsion stabilized by 1 wt% SDS can stay fairly stable up to 3 months. Similarly, Kumar and Mahto [55] reported that crude oil/water emulsions stabilized by ≥2 wt% 4–(1,1,3,3–tetramethylbutyl)phenyl-polyethylene glycol (a nonionic surfactant) were stable for 56 days. 

### 3.6. pH-Induced Destabilization of the Extremely Stable Nanoemulsions 

The long-term stability is evident from the result presented in Figure 6 even at as low SDBS concentration as 0.1 wt%. Although many industrial applications require the formulation of extremely stable nanoemulsions (such as the ones presented in this study), it will be even more desirable to easily break these highly stable nanoemulsions whenever needed. Currently, there are a number of demulsification processes (either physical or chemical) that might be applied to destabilize emulsions [56,57,58,59,60]; however, the demand in oil industry for a simple and, at the same time, effective demulsification method is still high. Such a simple and effective demulsification method is still lacking in the published literature. Therefore, we investigate herein the potential of pH alteration process (e.g., pH-switching) in breaking these extremely stable crude O/W nanoemulsions stabilized by SDBS, which does not possess a pH-responsive character.

Figure 6 shows the breakdown of a crude O/W nanoemulsion stabilized by 0.1% SDBS via the switching of the emulsion pH to a higher (using NaOH) or a lower (using HCl) value. As depicted in this figure, a complete breakdown (i.e., oil separation) is attained within 1 h when 0.5 M HCl was added. This result is similar to those reported for emulsions stabilized using emulsifiers possessing pH-responsive characters [14,35,61,62]. Given that the pKa value of SDBS is −6.5 [63] and since the final pH of the emulsion upon the addition of 0.5 M HCl is about 0.3, the surfactant molecules before and after the addition of HCl are negatively charged. Therefore, it might be, intuitively, expected that even after the addition of HCl, the negatively charged SDBS molecules would still repel each other and, thus, the emulsion would remain stable. However, since there is no change in the protonation state of SDBS, the demulsification induced by the addition of HCl (i.e., pH alteration) is quite surprising. It has been reported in the literature that SDS (belongs to the same family as SDBS) and also other sodium primary alkyl sulfates undergo acid-catalyzed hydrolysis, particularly when present at high concentrations (e.g., micellar solutions) [38,39,40]. Therefore, the pH-induced demulsification of the crude O/W nanoemulsion stabilized by 0.1 wt% SDBS (this concentration is above the surfactant CMC) might be attributed to the hydrolysis of the surfactant molecules. This hydrolysis could lead to the depletion of the surfactant molecules from the oil droplet interface and, accordingly, droplet coalescence and emulsion destabilization. Although this might be a contributing factor towards the complete demulsification and oil separation, it seems to not be the only driving force for such a quick and a complete demulsification as will be discussed subsequently.

Figure 6 also shows that the addition of 0.5 M NaOH results in a complete demulsification within 1 h, which is identical to the case of HCl addition. Arguably, SDBS molecules might still experience some hydrolysis at high pH values due to their existence in concentrated (micellar) surfactant solutions [38,39,40]. However, the long-term stability (in the absence of NaOH or HCl) of the nanoemulsions despite their relatively high SDBS contents (i.e., above CMC) rules out the likelihood of demulsification driven by SDBS concentration-related hydrolysis. Additionally, SDBS hydrolysis catalyzed by alkaline media is also unlikely to be significant and has not been reported in the literature to the best of our knowledge. Therefore, the existence of another demulsification mechanism (aside from the SDBS hydrolysis, if any) is very likely. This assertion is partially supported by the identically quick and complete demulsification of the crude O/W nanoemulsion stabilized by 0.1 wt% SDBS upon the addition of 0.5 M of either HCl or NaOH despite the expected significant differences between SDBS hydrolysis in acidic and basic media (if any). We, therefore, hypothesize that since crude oil (unlike the case of simple oils) contains some surface-active molecules (i.e., indigenous surfactants), these molecules might also adsorb at the surface of the oil droplets. The addition of HCl or NaOH seems to trigger some interactions, resulting in the depletion of SDBS (and probably some other indigenous surfactants) from the interface between the dispersed oil droplets and the continuous phase. The decreased interfacial density of SDBS could promote the coalescence of the oil droplets, which would consequently lead to demulsification.

The above hypothesis is supported by some findings reported in other studies. For instance, Ren and Zhang [64] found that chitosan is unable to stabilize Pickering emulsions under acidic media but when it was combined with SDS, the SDB–chitosan complex was able to produce stable Pickering emulsions under acidic media. Additionally, when hexadecyltrimethylammonium bromide (CTAB) was added to the Pickering emulsion stabilized by the SDS–chitosan complex, a quick demulsification was obtained. In another study, Liu et al. [65] reported that a Pickering emulsion formed from *n*-decane (or toluene), water, and negatively charged silica nanoparticles was not pH-responsive but when a trace amount of dodecyl dimethyl carboxyl betaine (a zwitterionic surfactant) was added, pH-responsiveness was observed, presumably, due to the interaction between the dodecyl dimethyl carboxyl betaine and the silica nanoparticles [65]. Pickering foams with a pH-responsive character were also obtained upon combining silica nanoparticles with a trace amount of dodecyl dimethyl carboxyl betaine [66]. Owing to the presence of some surface-active components in crude oil, the complete oil separation (i.e., 100% demulsification) from the O/W nanoemulsions stabilized by 0.1 wt% SDBS within 1 upon the addition of HCl or NaOH might stem, at least partially, from the depletion of SDBS from the surface of the oil droplets, driven by unfavorable interactions at highly acidic/basic media between SDBS and the indigenous surfactants in the crude oil. 

If the above hypothetical reasoning is likely true, the rate/extent of demulsification will be compromised by increasing the SDBS/crude oil ratio (i.e., SDBS/indigenous surfactant ratio). Increasing SDBS concentration while keeping the crude oil content unchanged will drive more SDBS molecules to the interface between the dispersed oil droplets and the surrounding continuous phase. This is, indeed, the case as suggested by the IFT, zeta potential, and droplet size results presented and discussed previously. Increasing the SDBS/indigenous surfactant ratio would eventually make the interfacial amount of SDBS at the droplet interface in excess of what is going to interact with the fixed indigenous surfactants. Ultimately, the excess amount of SDBS at the droplet interface would prevent the calescence, at least to a certain degree, resulting in incomplete oil separation upon the addition of NaOH or HCl. 

**Figure 7:**
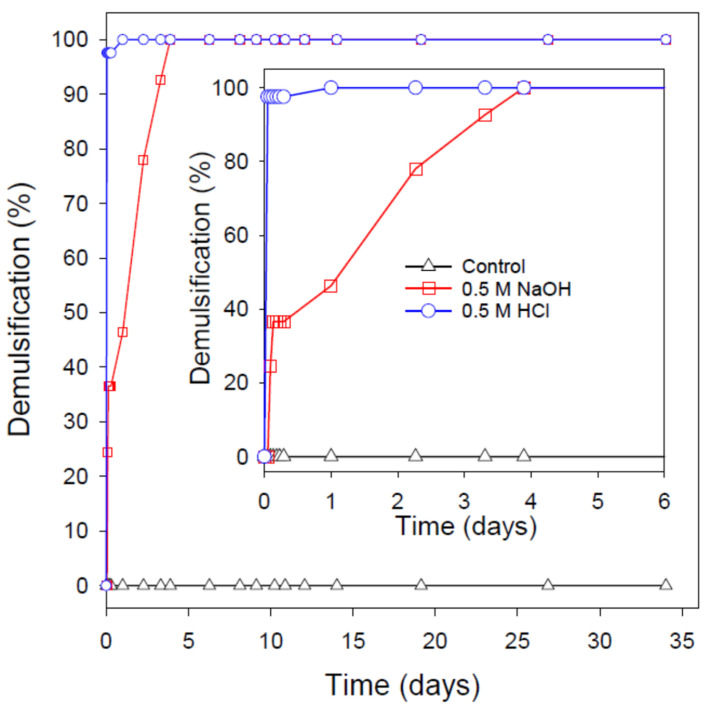
The long-term stability and demulsification process of the crude O/W nanoemulsion stabilized by 0.5 wt% SDBS using the pH-switching mechanism through the addition of 0.5 M HCl or NaOH. The demulsification process was conducted at ambient conditions and in the absence of any physical disturbances. The first 6 days of the demulsification process is shown in the inset.

**Figure 8:**
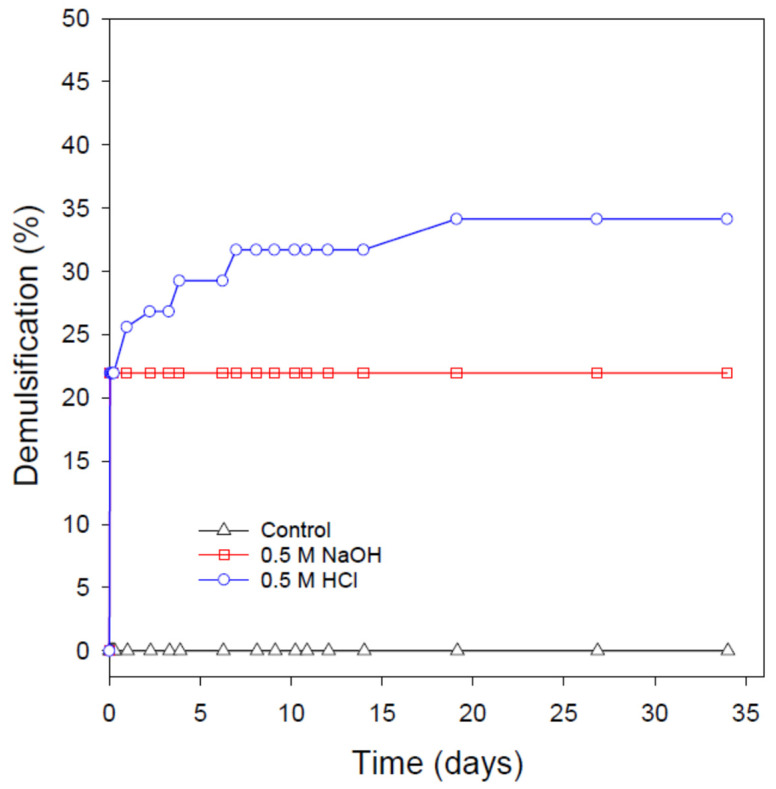
The long-term stability and demulsification process of the crude O/W nanoemulsion stabilized by 4 wt% SDBS using the pH-switching mechanism through the addition of 0.5 M HCl or NaOH. The demulsification process was conducted at ambient conditions and in the absence of any physical disturbances.

This is, in fact, the case as demonstrated in Figure 7 and Figure 8. Upon increasing the SDBS concentration in the emulsion to 0.5 wt%, a slower demulsification rate was observed even though a complete demulsification was eventually attained after about 1 and 4 days from the addition of HCl and NaOH, respectively (note: upon the addition of HCl, about 97.5% of the original oil in the emulsion was separated within 1 h, followed by a slow demulsification until a complete oil separation was achieved after 24 h). When the SDBS concentration in the emulsion was increased to the highest concentration used in this study (i.e., 4 wt%), the addition of NaOH and HCl resulted in only about 22 and 34% demulsification, respectively, even after storing the nanoemulsions for more than a month after the NaOH/HCl addition. This finding is in line with the demulsification of O/W emulsion stabilized by rhamnolipid biosurfactant, where the demulsification through the addition of NaOH was biosurfactant concentration-dependent [14]. However, based on the reasoning mentioned above, the pH-switching demulsification would depend on the nature of the oil. This is probably why a kerosene/water emulsion stabilized by surfactin (a biosurfactant with pH-responsiveness) was insensitive to NaOH addition [61].

## 4. Conclusions

Extremely stable crude oil/water nanoemulsions with excellent characteristics can be formulated using as low SDBS concentration as 0.1 wt%. Despite the extreme stability of the SDBS-stabilized nanoemulsions, they can be switched off (i.e., broken-down) via pH alternation using either HCl or NaOH despite SDBS not being a pH-responsive surfactant. The demulsification rate and extent, however, depend on the utilized SDBS dosage; a complete and a quick oil separation is attainable at SDBS dosages of 0.5 wt% and below. The long-term stability and the ease and simplicity of demulsifying these stable nanoemulsions are appealing. The nanoemulsion stabilized by 4 wt% SDBS is probably more attractive from an application standpoint due to its very small droplet size (as low 63.5 nm), lower IFT (as low as 0.45 mN/m), and the highly negative zeta potential (i.e., −62.2 mV). However, if the complete demulsification of this nanoemulsion in a later stage of the process via the proposed simple and cheap pH alteration method is required, it might not be a good candidate. Accordingly, the nanoemulsion stabilized by 0.5 wt% SDBS might stand out, given its reasonably low droplet size and zeta potential, long-term stability, and complete demulsification via the pH-switching mechanism. The findings reported herein open the doors for follow-up studies on tailoring this system (as well as other systems with different oils) for specific applications in oilfield industries such EOR, well-bore cleaning, well stimulation/remediation, and formation fracturing, among other oilfield-related applications.
